# The feasibility, acceptability, safety, and effects of early weight bearing in humeral fractures – a scoping review

**DOI:** 10.1080/09638288.2024.2351594

**Published:** 2024-05-16

**Authors:** Jia Hui Gan, Lindsay Bearne, Samuel Walters, Jon Room, Greg Booth, Alex Trompeter, Dimitra Nikoletou

**Affiliations:** a Haslemere Community Hospital, Royal Surrey NHS Foundation Trust, Surrey, UK; b Population Health Research Institute, St George’s University of London, London, UK; c Institute of Medical and Biomedical Education, St George’s University of London, London, UK; d Department of Trauma and Orthopaedic Surgery, St George’s University Hospitals NHS Foundation Trust, London, UK; e Faculty of Health and Life Sciences, Oxford Brookes University, Oxford, UK; f Physiotherapy Research Unit, Oxford University Hospitals NHS Foundation Trust, Oxford, UK; g Therapies Department, Royal National Orthopaedic Hospital Trust, London, UK

**Keywords:** Scoping review, humeral fractures, early weight bearing, immediate weight bearing, rehabilitation

## Abstract

**Purpose:**

Non-weight bearing is often recommended after humeral fractures. This review aims to summarise the extent and nature of the evidence for the feasibility, acceptability, safety, and effects of early weight bearing (EWB) in people with humeral fractures, treated operatively or non-operatively.

**Methods:**

Data sources identified published (PUBMED, EMBASE, CINAHL) and unpublished (ClinicalTrials.gov, CENTRAL, NIHR Open Research, OpenGrey) literature. Independent data extraction was conducted by two reviewers.

**Results:**

13 901 records were retrieved. Ten studies, involving 515 post-operative patients and 351 healthcare professionals, were included. EWB was found to be feasible in nine studies. There was limited evidence regarding adherence to EWB. Trauma and orthopaedic surgeons reported that EWB was acceptable. This depended on surgery type and whether it was a post-operative polytrauma case. No acceptability data was reported from patients’ perspectives. Only one study reported two patients who developed unsatisfactory outcomes from excessive post-operative EWB. Positive effects of EWB were reported on disability level, pain, shoulder and elbow motion, and union.

**Conclusion:**

There is some evidence for the feasibility, safety, and effectiveness of post-operative EWB after humeral fractures. There was limited data on the acceptability of EWB. Heterogeneous study designs, and variations in EWB protocols limit conclusions.

## IMPLICATIONS FOR REHABILITATION

- There is some evidence to support the feasibility, safety, and effectiveness of early weight bearing following operative management of humeral fractures.
- Early weight bearing after some humeral fractures is acceptable to some subspecialities of orthopaedic surgeons but is not universally accepted.
- Rehabilitation professionals should discuss the option of early weight bearing after operative management of humeral fracture with patients and their multidisciplinary team.

## Introduction

Humeral fractures are debilitating injuries that impact quality of life, particularly among older adults [1,2]. The global annual incidence of humeral fractures is increasing and currently represents 6–8% of all fractures in adults [3–5]. Humeral fractures can be classified based on their location within the bone as: proximal humerus, humeral shaft, and distal humerus [6–9]. These fractures can be stabilised operatively or nonoperatively. Nonoperative approaches are now standard of care for non-complex fractures and in older people [10–13]. However, rehabilitation after humeral fracture remains contentious. Typically, after injury, whether treated either operatively or non-operatively, a period of non-weight bearing (NWB) is prescribed. Recently, early weight bearing (EWB) is recognised as an underused rehabilitation treatment that has shown favourable outcomes [14,15].

At cellular level, bone healing after a fracture occurs in the presence of suitable external mechanical loading and local interfragmentary motion (strain) between fracture fragments [16–19]. By the second week post fracture, granulation tissue formation provides provisional stability before further bony bridging callus formation, leading to clinical union [20]. The adaptation of fractured bones to functional loading *via* EWB is integral to secondary healing but is underused in clinical practice [21,22]. This cautious approach to EWB may be because, during acute healing, a strategy is required that balances the benefits of EWB as mechanical stimulation with the potential risks of displacement in unstable fractures [20]. Excessive WB, beyond the therapeutic bandwidth of osteosynthesis, could potentially delay healing or cause non-union [23,24]. However, extended NWB is also associated with detrimental physiological and systemic complications and in older people could lead to loss of independence [25–30].

According to the British Orthopaedic Association Standards for Trauma and Orthopaedics (BOAST), all surgery in patients with frailty should be performed to allow full WB for activities of daily living within 36 hours of admission [31,32]. Traditionally, in humeral fractures, a minimum of six-weeks of non-weight bearing is regarded as the gold standard to promote healing, although this duration can vary and extend up to 12-weeks [33–35]. In the management of lower limb fractures, EWB has demonstrated numerous advantages and is now routinely applied; however, this is not yet the case after humeral fractures [36–39]. Additionally, it is recognised that the major stressors on the humerus are rotational force, which are different from the primary stressors of lower limbs – which are axial (force acting in the direction parallel to the axis of a bone) and bending forces during full weight bearing [40–42]. This evidence suggests that EWB following humeral fractures could be safer than weight bearing after a lower limb fracture.

No current or ongoing systematic or scoping reviews are being conducted on this topic [43]. The aim of this scoping review was to identify and summarise the existing research evidence on the feasibility, acceptability, safety, and effects of EWB in humeral fractures treated operatively or non-operatively.

## Methods

This scoping review was informed by the Joanna Briggs Institute for conducting scoping reviews and reporting, using the Preferred Reporting Items for Systematic Reviews and Meta-Analyses (PRISMA) Extension for scoping reviews [44,45]. The protocol was initially created on the Open Science Framework on 22 November 2022. Registration of the final protocol was 24 March 2023 (https://osf.io/zkad9/).

### Search strategy

Following piloting of the search strategy on PubMed and CINAHL Plus, seven electronic databases were searched for published or unpublished reports from 01 January 2000 to 20 March 2023 (Table 1). A systematic search strategy was designed that expanded the terms “humerus,” “weight bearing,” “fracture,” “operative,” “non-operative,” “rehabilitation” (Supplementary Data 1). The reference list of the studies included in the review were screened for additional potentially eligible records.

**Table 1.** Source of electronic databases.

Electronic databases	Electronic databases on grey literature websites
PubMed	ClinicalTrials.gov https://clinicaltrials.gov/
CINAHL Plus	Cochrane Central Register of Controlled Trials (CENTRAL) https://www.cochranelibrary.com/central/about-central
Embase	NIHR Open Research https://openresearch.nihr.ac.uk/?utm_source=google&utm_medium=sem&utm_campaign=JRH30302&gclid=Cj0KCQjwyOuYBhCGARIsAIdGQRNxOGDowiox2Tryrooj1eZEgUe9F5PenGcnKWFdCa3WXhMUJrFVUHMaApciEALw_wcB
	OpenGrey.EU https://opengrey.eu/

### Eligibility criteria

Eligibility criteria were characterised as participants, concepts, context, study types and outcomes (Table 2).

**Table 2.** Eligibility criteria.

	Inclusion criteria	Exclusion criteria
I. Participants	Adults who are at least 18 years old and have been diagnosed with humeral fractures through radiography.	Humeral fractures caused by pathological conditions. Individuals below 18 years old, and animal studies.
II. Concept	Studies investigating weight bearing within six weeks of post-humeral fractures [34].	Studies that do not include any weight bearing parameters.
III. Context	Studies published in English, between 1 January 2000 and 20 March 2023 [46].	Studies published in languages other than English and before 1 January 2000.
IV. Study types and designs	All full text research articles and grey literature sources.	Research article, conference abstracts and proceedings that cannot be retrieved in full-text after contacting the presenters or authors.
V. Outcomes	Studies that have reported parameters that measures feasibility, acceptability, safety, and effects of EWB in humeral fractures.	Studies that do not report any parameters that measures feasibility, acceptability, safety, and effects of EWB.

### Source of evidence selection

All records were imported into Mendeley reference management software (v2.93.0) and duplicates were removed [47], before being imported into Rayyan reference management [48]. Title and abstracts and then full-text were screened independently by at least two of four reviewers [JHG, SW, JR and GB]. Any discrepancies were resolved through discussions. In cases of uncertainty, the opinion of a third reviewer [LB, AT, DN] was sought to reach a consensus [49].

### Data extraction and charting

A data extraction template was adapted from the Joanna Briggs Institute System for the Unified Management, Assessment, and Review of Information, *a priori* and piloted with five articles [50]. Key study characteristics collected included participant’s characteristic, study’s characteristics, characteristic of humeral fractures and its management, WB protocol and parameters, outcomes of EWB in feasibility, acceptability, safety, and effects. One reviewer [JHG] extracted data from all included studies. A second reviewer [GB] independently extracted data from 50% of the included studies and any discrepancies were discussed. Finally, the third reviewer [DN] cross-checked 10% of the extracted data for accuracy by comparing it against the full-text articles. If necessary, the reviewers contacted the corresponding authors of included studies to clarify any missing or additional data.

### Data synthesis and analysis

Study characteristics and outcomes of interest were summarised using descriptive statistics (e.g., percentages, mean, standard deviation and range) and narratively to describe the nature and extent of the evidence for each outcome of interest. The outcomes specific to address the research question of this study underwent quantitative analysis to identify the existing literature gaps.

## Results

### Study identification

**Figure 1.**
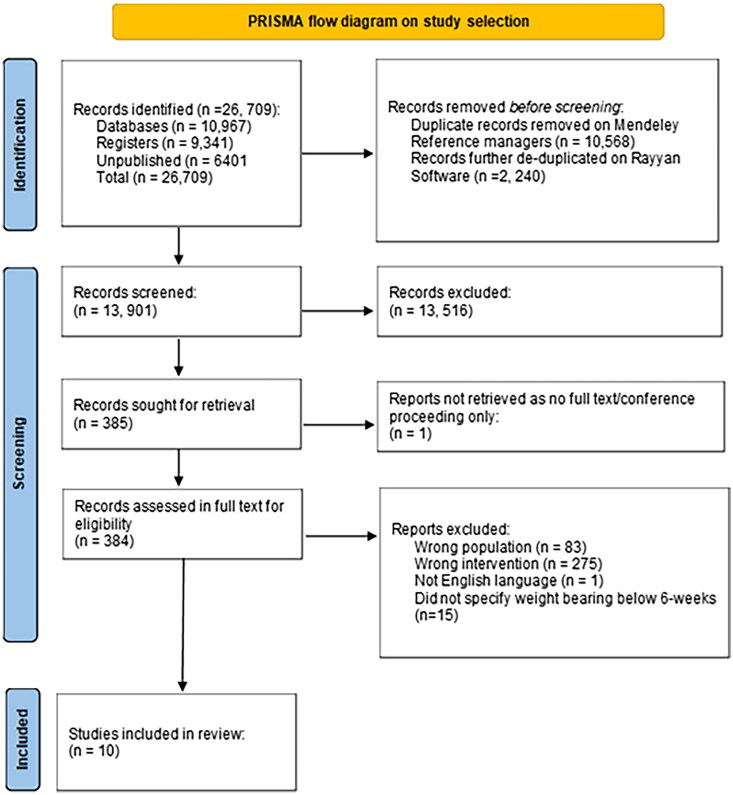
PRISMA flow diagram for study selection for this scoping review.

A total of 26 709 citations were identified. After duplicates were removed, 13 901 records were retrieved and full texts of 385 articles were screened. 10 studies were included in the final analysis (Figure 1 and Supplementary Data 2).

### Studies included and participants characteristics

Studies included three retrospective cohort studies [51–53], two case series [54,55], two cross sectional surveys [56,57], one commentary article [58], one retrospective case series [59], and one prospective case series [60] (Table 3).

**Table 3.** Study and participant characteristics .

Study	Study characteristics	Location of humerus fracture	Classification on types and number of fractures	Sample size (n)	Mean age and range of participants (years)	Gender of participants	Country of participants	Presence of comorbidities	Duration of follow-up (weeks)	Type of fracture treatments	Other rehabilitation treatments
Ayoub and Tarkin [58]	Commentary article	Distal humerus fractures	Extra-articular; not retrievable (NR) on number.	NR	NR	NR	USA	NR	NR	Operative (NS) and non-operative	NR
James et al. [54]	Case series	Proximal and humeral shaft fractures	Proximal metadiaphyseal humerus fragility fracture, NR on number.	18	70; range NR	NS	USA	Rotator cuff injury.	Mean 134.7 weeks (range 52–182.5 weeks)	Operative (ORIF)	Sling, physiotherapy
Langhammer et al. [51]	Retrospective cohort	Humeral shaft fractures	Orthopaedic Trauma Association (OTA) fracture type: A1 (*n* = 20), A2 (*n* = 40), A3 (*n* = 77), B1 (*n* = 12), B2 (*n* = 53), B3 (*n* = 17), C1 (*n* = 6), C2 (*n* = 8), C3 (*n* = 8).	241	38; range 14–88	73 females, 163 males	USA	NR	Mean 42.8 weeks (range 3–456 weeks)	Operative (ORIF with plates)	NR
Mayer et al. [55]	Case series reports (four case studies labelled as case study i, ii, iii, and iv	Distal humerus fractures	i. Type C comminuted metadiaphyseal and articular segments. ii. Osteopenia with a complex articular injury with multiple chondrocancellous pieces and intercalary comminution. iii. Complex articular injury with capitellar and trochlear shear fractures. iv. NR.	4	4; range 60–77	3 females, 1 male	USA	Sacral fracture, distal radius fracture, osteogenesis imperfecta, childhood chronic elbow instability with deformity.	Mean 47.0 weeks (range 12.0–104.0 weeks)	Operative (total elbow arthroplasty)	NR
Patch et al. [56]	Cross sectional survey	Proximal humerus fractures	NR	293	NR	NR	USA	NR	NR	Operative (ORIF or shoulder arthroplasty)	Various rehabilitative patient education method, formal outpatient, passive range of motion (ROM) exercises.
Sharareh and Perkins [57]	Cross sectional survey	Humeral shaft fractures	Diaphyseal, NR.	58	NR	NR	USA	NR	NR	Operative (NS) and non-operative	NR
Stephens et al. [59]	Retrospective case series	Distal humerus fractures	Comminuted fractures, deemed non-reconstructable by Open Reduction and Internal Fixation (ORIF); NR.	9	71; range 55–92	NR	USA	Using mobility aids, osteoporosis, coronary artery disease, atrial fibrillation, distal radius fracture.	mean 191.6 weeks (range 100–417.2 weeks)	Operative (elbow hemiarthroplasty)	Sling, active assisted ROM exercises, strengthening exercises.
Suzuki et al. [52]	Retrospective analysis of prospective database	Humeral shaft fractures	OTA classifications: 12 A (*n* = 7), 12B (*n* = 5), 12 C (*n* = 5); open fractures (*n* = 10).	17	33.5, range NR	8 females, 9 males	USA	Radial nerve palsy, below elbow complete nerve deficits, traumatic amputation at the distal third of humerus, prolonged unconsciousness.	NR	Operative	ROM exercises
Szczęsny et al. [53]	Retrospective cohort study	Proximal humerus fractures	Comminuted fractures with least three-part of Neer’s fractures; NR on number.	2	NS	NR	Poland	Unspecified mental disturbance, myocardial infarction.	Mean 75.6 weeks (range 52–260.2 weeks)	Operative (ORIF)	NR
Wajnsztejn et al. [60]	Prospective case series	Distal humerus fractures	*Arbeitsgemeinschaft für Osteosynthesefragen* (AO) / OTA types of all B or C; NR on number.	13	39; range 22–68	4 females, 9 males	Brazil	NR	Mean 69.5 weeks (range 52–87 weeks)	Operative (ORIF)	Neither sling nor orthotic used; passive ROM exercises, active ROM exercises.

AO: *Arbeitsgemeinschaft für Osteosynthesefragen*; DASH: Disabilities of the Arm, Shoulder, and Hand questionnaire; EWB: early weight bearing; NR: not retrievable; NS: not specified; NWB: non-weight bearing; ORIF: open reduction and internal fixation; OTA: Orthopaedic Trauma Association; ROM: range of motion; TEA: total elbow arthroplasty; USA: United State of America; WB: weight bearing.

Most of the studies (eight studies, 80%) were conducted in the United State of America [51,52,54–59], with one study each conducted in Europe (Poland) [53] and, South America (Brazil) [60]. Six studies reported retrospective longitudinal data. The mean study duration was 93.5 weeks (range 3.0–456.0 weeks) [51,53–55,59,60].

Nine studies included a total of 515 patients with humeral fractures and 351 healthcare professionals [136 trauma and orthopaedic surgeons, 172 shoulder surgeons]) [51–57,59,60]. The mean patient age was 39.3 years (range 22 to 92 years) (6 studies, 284 patients) [51,52,54,55,59,60]. Sixty-seven % patients were males (report in only four studies, 270 patients) [51,52,55,60].

Five studies reported patient comorbidities including rotator cuff injuries, other sites of fractures, congenital genetic diseases, required assistive walking aids, osteoporosis, cardiac disease, traumatic amputation, prolonged unconsciousness, and unspecified mental disturbance [52–55,59]. Five studies did not report patient comorbidities [51,56–58,60].

### Fracture classifications and management

Three studies focused on fractures of the humeral shaft [51,52,57], and four studies investigated distal humeral fractures [55,58–60]. Two studies investigated proximal humeral fractures [53,56]. One study investigated management of proximal metadiaphyseal humeral fractures which is an infrequent severe fracture involving both the proximal and humeral shaft (Table 4) [54].

**Table 4.** Feasibility, acceptability, safety and effects of fractures and weight bearing related management.

Study and type of humeral fractures	Weight bearing protocol	Type of weight bearing instructions	Feasibility	Acceptability	Safety (adverse event)		Effects	
					EWB specific	Unspecific to EWB	EWB specific	Unspecific to EWB
Ayoub and Tarkin [58]; distal humeral fractures	Immediate WB	NR	Facilitator: (+), operation in polytrauma.	Surgeons: accepted.	NR	I. nonoperative cases: nonunion rate of 12–15%, malunion deformity in non-operative treatment rate of 18% −28%, and secondary operation due to non-union rate of 10%. II. operative case: no patient had more than 5-degree angulation deformity.	Functional outcomes: allowed immediate ambulation with WB on walking aids for rehabilitation	Union: I. Nonoperative cases unreliable data from high loss to follow-up of a single study. II. Operative cases: 95–100% of union rate in operative cases. Clinical outcomes: I. Nonoperative cases: 45% of patients had a decreased in shoulder external rotation and 24% loss of elbow extension. II. Operative cases: 90% patient satisfaction Functional outcomes: I. Nonoperative cases: 50% of patients had excellent functional outcomes. II. Operative cases: earlier rehabilitation, active resumption and return to work.
James et al. [54]; proximal metadiaphyseal humerus fractures	Immediate WB	Use of arms allowed for ADLs with a weight limit of 10 pounds.	NR	NR	NR	5% of Radial nerve palsy	NR	Union: uneventful union in all cases mean = 135.7 weeks (range 52 − 182.5) [1 was unable to assess due to death from unrelated causes before healing can be objectively measured]. Clinical outcomes: DASH scored 12, (mean 21, standard deviation = 20); pain score in DASH question = 75% reported no pain, 25% reported no more than moderate pain. Functional outcomes: 75% reported shoulders achieved overhead functionality.
Langhammer et al. [51]; humeral shaft fractures	Immediate WB for 53% of participants; NWB for 47% of participants	WB as tolerated	Facilitator: (+), operation. Adherence: queried on treatment fidelity in performing immediate WB.	Surgeons: accepted.	NR	10% of Radial nerve palsy, 7% of nonunion in operative cases.	NR	Union: union was achieved in 225 fractures (93%) following operative treatment. Union was achieved in 117 of the 127 patients (92%) who were made WB as tolerated and 108 of the 114 patients (95%) who were made non-weight bearing (NWB). Clinical outcomes: NR Functional outcomes: NR
Mayer et al. [55]; distal humeral shaft fractures	Immediate WB	Immediate WB	Barrier: (+), older person reliant on upper limbs as WB limbs created additional demand on surgical im-plants.	Surgeons: accepted.	NR	25% of elbow healed with heterotopic bone formation (malunion) with elbow flexion from 20 to 120 degrees.	NR	Union: all four cases achieved radiographically and clinical healing between 12 and 26 weeks (six months). Clinical outcomes: elbow’s range of motion (ROM) 5–120 degrees (range 20 −1 05 degrees), supination pronation arc of 120 degree Functional outcomes: ambulated without walking aid at six-months.
Patch et al. [56]; proximal humerus fractures	Immediate WB or NWB	NR	Barriers: (+), decision to EWB by surgeons was driven by type of surgery, professional experience, patient characteristics, and fracture characteristics.	Trauma and orthopaedic surgeons: accepted. Shoulder surgeons: not accepted. Fellowship trained surgeons: not accepted.	NR		NR	NR
Sharareh and Perkins [57]; humeral shaft fractures	EWB, NS on timeframe	NR	Facilitator: (+), operation in polytrauma.	Trauma and orthopaedic surgeons: accepted.	NR		NR	NR
Stephens et al. [59]; distal humerus fractures	Immediate WB	WB as tolerated	Facilitator: (+) Elbow hemiarthro-plasty permits EWB for both younger and older persons than total elbow arthroplasty. Adherence: queried of treatment fidelity in performing immediate WB.	NR	NR	11% required revision orthopaedic surgery, required haematoma evacuation surgery and ulnar nerve transposition surgery.	NR	Union: NR Clinical outcomes: Mayo elbow performance score (MEPS): 76.1 points as fair outcomes); Patient rated elbow extension (PREE) of 39.66 points as fair outcomes; subjective pain score was moderate in MEPS. Functional outcomes: patients reported overall preserved functional capabilities with average PREE scored 39.66 and average MEPS scored at 70 at 44.1 months.
Suzuki et al. [52]; humeral shaft fractures	Immediate WB	Immediate WB	NR	Surgeons: accepted.	NR	No apparent adverse outcomes	NR	Union: bony unions with average healing time was 11.1 weeks (range 8–14 weeks) Clinical outcomes: NR Functional outcomes: NR
Szczęsny et al. [53]; proximal humerus fractures	NR	Avoid overloading of the extremity postoperatively	Barrier: two patients had physical and mental incapacity as in concurrent acute lower limbs amputation, removal of a hip prothesis from septic loosening, and postoperative mental disturbance. Adherence: (−), two out of 131 patients did not adhere to safety precaution of WB postoperatively.		Humeral Tubercles fragmentation and secondary dislocation.	12% fixation failures, 1.5% humeral head necrosis, 0.7% pathological fracture, and 0.3% ostensible false stabilisation from inappropriate patient positioning for X-rays radiography.	NR	NR
Wajnsztejn et al. [60]; distal humerus fractures	Immediate WB	Immediate loading	NR	Surgeons: (+)	NR	1 patient developed neuropraxia, and 1 patient developed superficial infection that required oral antibiotic and local debridement.	NR	Union: all patients had fracture healed within 12-weeks. Clinical and functional outcomes: DASH scored 0 (best functions possible) in 38% patients.

Abbreviations: ADLs: activities of daily living; NR: not retrievable; NS: not specified; EWB: early weight bearing; NWB: non-weight bearing; ROM: range of motion; WB: weight bearing; (+): positive related to early weight bearing; (−): negative related to early weight bearing.

All studies included patients who were managed operatively following their humeral fractures (Table 3). Two studies did not specify the operative approach [57,58]. Six studies used open reduction and internal fixation (ORIF) which included screws, plates, neutralisation devices, and external fixation then planned conversion to ORIF [51,53,54,56,60,61]. Three studies employed arthroplasty techniques, that including of shoulder arthroplasty, total elbow arthroplasty and elbow hemiarthroplasty [55,56,59]. One study explored the preference of EWB after shoulder arthroplasty or ORIF among shoulder, and trauma and orthopaedic surgeons [56]. However, there was a lack of detailed information about the reason surgeons preferred EWB after shoulder arthroplasty versus ORIF. No studies discussed the use of EWB after intramedullary nailing. Only one study that used different type of ORIF reported no relationship between nonunion and postoperative WB status [51].

Only two studies discussed nonoperative approaches as a possible first line treatment following midshaft and distal humeral fractures, including splinting, commercial or functional bracing [57,58]. One study specifically advocated for operative treatment over non-operative management, particularly in younger patients with distal humeral fractures [58].

Five studies provided information on post-operative rehabilitation [52,54,56,59,60]. This included the use of a sling [54,59], no sling nor orthotic [60], patient education [56], outpatient follow-up [56], unspecified physiotherapy programme [54], and home exercise programme [56]. Four studies detailed mobilisation exercises [52,56,59,60], but these varied in the timing of commencement and type of starting point, ranging from passive range of motion (ROM), active assisted ROM, and active ROM before strengthening exercises [52,56,59,60].

These three studies used validated outcome measures including the Disabilities of the Arm, Shoulder, and Hand (DASH) questionnaire [62], Patient Rated Elbow Evaluation (PREE) [63], and Mayo Elbow Performance Score (MEPS) [54,59,60,64]. Whereas, the other seven studies reported non standardised outcome measures.

### Early weight bearing protocol in humeral fractures

Two common postoperative EWB protocols were identified. Eight studies investigated WB immediately after surgery [51,52,54–56,58–60]. One study mentioned EWB without specifying a timeframe [57], whilst another study did not specify the terminology of EWB but advised patients to use their arm but “avoid overloading the extremity postoperatively” which was categorised as EWB (Table 4) [53]. In two studies that used immediate WB protocols, patients were further instructed to “weight bear as tolerated” [51,59]. Only one study provided more specific instructions, allowing arm use for daily activities but restricting lifting to 10-pound or less [54]. However, the timeframe for EWB was not explicitly defined in any included studies.

### The feasibility of early weight bearing

The feasibility of EWB was reported in six studies, exploring the *facilitators, barriers*, and *adherence* to EWB (Table 4) [51,53,55,56,58,59]. Two studies highlighted that operative management functioned as a *facilitator* for EWB [57,58], especially in cases involving polytrauma [51,59]. Setting preoperative rehabilitation goals for prompt postoperative ambulation was also identified as a facilitator.

Three studies focused on *barriers* to EWB [53,55,56]. These barriers encompassed patient characteristics, surgeon’s experience, fracture characteristics, and the type of operative fixation. Some of these factors affected the patient’s ability to WB immediately and tolerate additional upper limb WB, such as during sit-to-stand or when using walking aids [55,56]. Szczęsny et al. specifically reported the characteristics of two patients with mental and physical incapacity. These patients developed postoperative delirium and had reduced ambulation due to bilateral amputation or the removal of a hip prothesis, which hindered their adherence to post-operative WB instructions [53].

Three studies briefly mentioned patient’s adherence to EWB instructions [51,53,59]. Two retrospective studies queried the patient’s adherence to the prescribed immediate WB protocols [51,59], although no further investigation was completed. Szczęsny et al. reported that two out of 131 patients did not adhere to the postoperative instruction to “avoid overloading,” although it is not clear whether this included the EWB protocols [53].

### The acceptability of early weight bearing

No studies investigated the acceptability of EWB from the patients’ perspective. The acceptability of EWB from the orthopaedic surgeon’s perspective was considered in 7 out of 10 studies [51,52,55–58,60]. Some surgeons perceived EWB as acceptable in certain instances, such as polytrauma, older people who required upper limbs to support the use of walking aids or patients who required expedient return to activities of daily living, employment, or an important social role [57–59]. However, there were no standardised postoperative EWB instructions. Instead, five studies emphasised that EWB protocols should be person centred and individualised [51,52,55,58,60] and consider pre-operative planning, the appropriate operative implants, and postoperative rehabilitation.

One study assessed the acceptability of EWB following postoperative proximal humeral fractures among different sub-specialities of orthopaedic surgeons [56]. It reported that a greater proportion of trauma and orthopaedic surgeons considered EWB acceptable after arthroplasty, but not ORIF, compared to shoulder surgeons [56]. Another survey was conducted exclusively amongst trauma and orthopaedic surgeons, and it found that they were more likely to recommend EWB in post-operative humeral shaft fractures and polytrauma cases [57].

### The safety of early weight bearing

The safety of EWB in the post-operative management of humeral fractures was reported in only five studies (Table 4) [51–53,55,60]. One study reported that EWB did not cause non-union in postoperative humeral shaft fractures [51]. Another study reported no adverse effects after EWB in post-operative distal humerus fractures without using slings or orthotics [60]. One study investigating EWB after post-operative comminuted proximal humeral fractures found that only two of 131 patients had secondary destabilisation following EWB [53]. These patients had post-operative delirium and lower limb disability and did not adhere to safety advice. The excessive WB resulted in poor operative outcomes, including humeral tubercles fragmentation and secondary dislocation [53]. It is worth noting that these patients had additional challenges postoperatively, which may have affected their cognitive ability to understand the risk of overloading a severe humeral fracture after surgery [53].

### The effects of early weight bearing

Five studies reported the overall positive effects of post-operative EWB following humeral fractures on disability level, pain, shoulder and elbow’s motion and functional outcomes [54,55,58–60]. Five studies reported fracture healing time and extent [51,52,54,55,60]. Three studies did not comment on the effects of EWB [53,56,57].

The studies that used standardised outcome measures such as DASH, PREE, MEPS to measure the effects of EWB all demonstrated improvements in disability levels [54,59,60]. For instance, Wajnsztejn et al. reported that 38% of patients achieved a score of 0 on the DASH scale (indicating the best possible outcome) [60], whilst James et al. reported mean DASH score of 21 (median 12; standard deviation 20) [54], meaning that their patients could be considered clinically indistinguishable from the general population. Stephens et al. rated the improvements as “fair” after EWB, (mean PREE score of 41 points, mean MEPS score of 76.1 points) [59]. Patients reported that their upper limbs’ functional capabilities were generally preserved at 44 months.

Only two studies reported the effect of EWB on pain [54,59]. One study reported that 75% of patients experienced no pain, whilst 25% reported no more than moderate pain measured by the DASH questionnaire [54]. Likewise, in another study, moderate average pain scores were measured by the MEPS [59].

Only three studies reported the effects of EWB on shoulder or elbow motion [54,55,58]. In one case series, three out of four patients achieved a maximum elbow range of motion from 5 to 120 degrees at mean (range) follow up of 47 (12–104) weeks [55]. Two studies used unspecified measures to assess range of motion but mentioned that post-operative upper limb usage for ambulation had improved, and another study reported that 75% of patients were able to lift their arms above their head at mean (range) 132 (52 to 182.5) weeks [54,58].

There was insufficient information to estimate the overall healing time of fractures in two out five studies [51,54,60]. However, James et al. [54] reported uneventful fracture healing in all 18 patients, whilst Langhammer et al. [51] stated that union was achieved in 93% of patients although there was no statistically significant relationship (*p* = 0.45) between postoperative WB status (EWB versus NWB) and union rate, regardless of the size of fixation plates used. Wajnsztejn et al. [60] defined fracture union as bridged cortices on 2 radiographic planes and absence of union during movement. This study reported that all patients (*n* = 13) who underwent immediate postoperative WB had fracture healing within 12 weeks. Two other studies reported bony healing on radiography after postoperative EWB [52,55]. Mayer et al. [55] (*n* = 4) reported initial radiographic healing at 12 weeks, with clinical and full radiographic healing at six months; Suzuki et al. [52] (*n* = 17) reported radiographic bony union at a mean (range) of time of 11 (8–14) weeks.

## Discussion

This review found that there was some evidence for the feasibility, safety, and effectiveness of EWB in humeral fractures following operative management. EWB after some surgical approaches is acceptable to some orthopaedic surgeons. However, research gaps exist, including the absence of investigations into EWB following non-operative management of humeral fractures, the optimal timeframe for EWB, and the acceptability of EWB in patients, their relatives, and the wider multidisciplinary healthcare team. The study populations included in our review are similar to the age distribution in epidemiological data for humeral fractures, ensuring the generalisability of our findings [3,6,65].

Only six studies examined the facilitators, barriers, and adherence in relation to the feasibility of EWB. Overall, the included studies indicate that setting pre-operative goals [51,57–59], especially in polytrauma cases, can help people’s mobility, consistent with findings of previous studies [10,66,67]. This finding is also evidenced by three studies that compared non-operative and operative fixation in patients with polytrauma and concomitant humeral fractures [68–70]. Our review highlighted that there were four main barriers to post-operative EWB after humeral fracture, this included patient characteristics, surgeon’s experience, fracture characteristics, and the type of operative fixation. These findings are similar to barriers reported in a study of EWB after hip fractures [71]. However, additional barriers to EWB were identified after hip fracture and this may include patient characteristics, increased operative time, pre-holiday surgery and admissions in the first quarter of the year. Patient characteristics, such as post-operative delirium, mental incapacity, and lack of lower limb from amputation are vital safety considerations when implementing EWB after humeral fractures [51,55,58].

Concerns regarding the feasibility and safety of applying EWB post-operatively were raised in two studies [51,59]. Both studies highlighted the risks associated with poor adherence to unexplicit EWB status in some patients [51,59]. These concerns were also raised in people following lower limb fractures, especially in older people [72]. In the non-operative management of displaced lower limb fractures (e.g., pelvic or acetabular fractures) these risks were addressed by adapting the EWB guidelines [73]. One study of post-operative EWB after hip fracture demonstrated that early EWB and mobilisation was feasible and had high adherence (78%) [74]. These EWB protocols were also feasible in patients with cognitive impairment or people with multi-comorbidities [74]. Therefore, post-operative EWB approach should be considered after humeral fractures if accompanied by clear rehabilitation instructions [75].

The acceptability of EWB following humeral fractures has only been investigated from the perspectives of surgeons. Only two included studies found that EWB post-operatively was more acceptable to trauma and orthopaedic surgeons compared to shoulder surgeons [56,57]. This preference was limited to arthroplasty surgery alone [56], perhaps due to the belief that absolute fracture stability is required to allow secondary healing for osteosynthesis. This was further influenced by surgeon’s subspecialty training in shoulder and elbow surgeries [56]. This preference is similar to a national audit of EWB following ankle fracture, where only 21% of operatively managed patients were recommended to EWB by surgeons contrary to clinical guidelines [76,77]. Whereas immediate WB following hip fractures with precautionary measures was successfully implemented as standard of care [78,79]. This provides an example of a successful clinical pathway for EWB that could be mirrored for post-operative humeral fractures. However, it is crucial to establish active collaborations among patients and their relatives and multi-disciplinary professionals to codesign evidence-based protocols and pathways, so they are feasible and acceptable [80].

Adverse events were seldom reported in our included studies. One study supported the application of EWB or immediate post-operative WB protocols following humeral fractures, particularly in people with frailty or polytrauma [53]. This is because humeral fractures can lead to substantial functional impairment and reduced health-related quality of life, that is compounded by NWB restrictions [34,81]. Langhammer et al. [51] found no link between immediate WB protocols and adverse events in post-operative humeral shaft fractures. Biomechanical studies also supported the safety of immediate WB post-operatively in humeral shaft and distal humeral fractures [82–84]. These biomedical studies suggest that the benefits of early rehabilitation and muscle strengthening from an immediate WB approach can enhance the performance of the surgical implants and patient outcomes [67.]

However, it is imperative that WB protocols are adhered to as additional postoperative fractures and secondary dislocation were reported in one study (two patients) following overloading [53]. Secondary displacements of proximal humeral fracture affect over 12.5%–28.8% of cases post-operatively [85,86]. To minimise this risk, Tingstad et al. suggested stratification of post-operative WB protocols following humerus fractures based on the presence of lower limb injury that required restricted WB restriction, rather than humeral fracture pattern or severity [87]. In addition, a multi-professional integrated care model involving biomechanical engineers and rehabilitation clinicians may help mitigate the risk of loading failure in an EWB rehabilitation pathway following a humeral fracture [71,88–91]. The findings from five included studies [51,52,54,55,60]. suggest that EWB has no deleterious effect on the union or malunion rate, which aligns with an early study of EWB in humeral shaft fractures [87]. These findings suggest that promoting independence through immediate or EWB protocols is warranted. Independence in social roles matters more for patient at high mortality risk, which is a stronger predictor of positive outcomes than age alone [92].

## Limitations and strengths of this scoping review

This review had several limitations. The small number of included studies, as well as the heterogeneity of patient demographics, operative methods, treatment, and WB protocols mean our findings should be interpreted with caution. These limitations hinder accurate implementation of EWB as part of the functional rehabilitation process, especially distinguishing between the therapeutic effects of primary and secondary bone healing. Additionally, the included retrospective cohort studies and case series, which do not have comparison groups, mean it was not possible to calculate between group effect sizes for each outcome. It was also not possible to stratify outcomes by EWB or immediate WB approaches for different type of humerus fractures, operative or non-operative management. This review, included comprehensive search strategy and followed a standardised framework [43]. Due to funding constraints, it was not possible to assign two reviewers to extract data from all evidence sources. Instead, one reviewer extracted all the data and a second reviewer extracted 50% of the sources. A third reviewer checked the extraction accuracy for 10% of randomly selected evidence sources thus minimising the risk of data inaccuracies [93].

## Future research recommendations

Future studies should develop standardised definitions and terminology for immediate and EWB when applied to the management of humeral fractures, build the evidence for the dosage, timeframes, and instructions for applying EWB protocols for both operative and non-operative management of humeral fractures and explore the perspectives of patients and healthcare professional about EWB protocols. While studies that evaluated the effect of post-operative EWB using standardised outcome measures demonstrated improvements in disability and function there is no core outcome set which makes further synthesis difficult [54,59,60]. The development of a core outcome set is needed for research into the effectiveness of EWB after humeral fracture [94,95].

## Conclusion

This scoping review revealed that there is some evidence for the feasibility, safety, and effectiveness of EWB in humeral fractures following operative management only. EWB after some surgical approaches are acceptable to some sub-speciality of orthopaedic surgeons, but this is not universal among orthopaedic surgeons. Robust research studies using a core outcome set are warranted to establish effective guidelines and clinical decision tools for the implementation of EWB after humeral fractures.

## Supplementary Material

Supplemental Material

## Data Availability

The data supporting the findings of this study are available from the corresponding author upon reasonable request.

## Data set

https://osf.io/zkad9/.
