# Supplementary material for: The feasibility, acceptability, safety, and effects of early weight bearing in humeral fractures – a scoping review
Source: Disabil Rehabil. 2024 May 16;47(3):519–30. doi: 10.1080/09638288.2024.2351594 (PMC11789713; doi:10.1080/09638288.2024.2351594)
Supplement: Supplemental Material [file IDRE_A_2351594_SM6464.zip › Databases and websites search.docx]

| **Databases** | **Results** |
| --- | --- |
| PubMed (Medline) | 8,698 |
| Embase (Ovid) | 10 |
| CINAHL Plus | 2,261 |
| Subtotal | 10,967 |
| **Websites for Grey Literature** | **Results** |
| ClinicalTrials.gov | 8,998 |
| 2. Cochrane Central Register of Controlled Trials (CENTRAL) | 106 |
| 3. NIHR Open Research | 237 |
| 4. OpenGrey.EU | 6,401 |
| Subtotal | 15,742 |
| **Total** | 26,709 |
| **Following removal of duplicates on Mendeley Reference Manager** | 16,141 articles |
| **Following removal of duplicates on Rayyan** | 13,901 articles |
| **13, 901 articles divided by 3 reviewers** | 4634 |
| **13, 901 articles divided by 4 reviewers** | 3476 |

# **1. Databases:**

| **Search location** | **Databases Search (date)** | **Limits** | **Results** |
| --- | --- | --- | --- |
| PubMed (Medline) | (("weight" OR "weight bearing" OR "immediate" OR "early" OR "avoid" OR "non" OR "loading" OR "patient guided") AND ((english[Filter]) AND (2000:2023[pdat]))) AND (("humerus" OR "humeral" OR "proximal" OR "midshaft" OR "distal" OR "arm") AND ((english[Filter]) AND (2000:2023[pdat]))) AND (("fracture" OR "fractures" OR "fractured" OR "broken") AND ((english[Filter]) AND (2000:2023[pdat]))) AND (("treatment" OR "surgical" OR "non surgical" OR "operative" OR "non operative" OR "rehabilitation") AND ((english[Filter]) AND (2000:2023[pdat]))) (20/03/23) | Start date: 01/01/2000  English language only | 8698 |
| Embase (Ovid) | See below for full search strategy (20/03/23) | Start date: 01/01/2000  English language only | 10 |
| CINAHL Plus | See below for full search strategy (20/03/23) | Start date: 01/01/2000  English language only | 2261 |

# **1.1 Databases: Embase (Ovid)**

# Searched on 20/03/2023.

1 weight bearing.mp. [mp=title, abstract, heading word, drug trade name, original title, device manufacturer, drug manufacturer, device trade name, keyword heading word, floating subheading word, candidate term word] 39727

2 limit 1 to (english language and yr="2000") 712

3 loading.mp. [mp=title, abstract, heading word, drug trade name, original title, device manufacturer, drug manufacturer, device trade name, keyword heading word, floating subheading word, candidate term word] 206528

4 limit 3 to (english language and yr="2000") 2426

5 2 or 4 3002

6 immediate.mp. [mp=title, abstract, heading word, drug trade name, original title, device manufacturer, drug manufacturer, device trade name, keyword heading word, floating subheading word, candidate term word] 262490

7 limit 6 to (english language and yr="2000") 4035

8 early.mp. [mp=title, abstract, heading word, drug trade name, original title, device manufacturer, drug manufacturer, device trade name, keyword heading word, floating subheading word, candidate term word] 2239249

9 limit 8 to (english language and yr="2000") 29201

10 avoid.mp. [mp=title, abstract, heading word, drug trade name, original title, device manufacturer, drug manufacturer, device trade name, keyword heading word, floating subheading word, candidate term word] 285542

11 limit 10 to (english language and yr="2000") 2965

12 patient guided.mp. [mp=title, abstract, heading word, drug trade name, original title, device manufacturer, drug manufacturer, device trade name, keyword heading word, floating subheading word, candidate term word] 126

13 limit 12 to (english language and yr="2000") 1

14 7 or 9 or 11 or 12 34802

15 humerus.mp. [mp=title, abstract, heading word, drug trade name, original title, device manufacturer, drug manufacturer, device trade name, keyword heading word, floating subheading word, candidate term word] 29506

16 limit 15 to (english language and yr="2000 - 2020") 404

17 humeral.mp. [mp=title, abstract, heading word, drug trade name, original title, device manufacturer, drug manufacturer, device trade name, keyword heading word, floating subheading word, candidate term word] 19310

18 limit 17 to (english language and yr="2000") 215

19 proximal.mp. [mp=title, abstract, heading word, drug trade name, original title, device manufacturer, drug manufacturer, device trade name, keyword heading word, floating subheading word, candidate term word] 271393

20 limit 19 to (english language and yr="2000") 4635

21 midshaft.mp. [mp=title, abstract, heading word, drug trade name, original title, device manufacturer, drug manufacturer, device trade name, keyword heading word, floating subheading word, candidate term word] 2770

22 limit 21 to (english language and yr="2000") 31

23 distal.mp. [mp=title, abstract, heading word, drug trade name, original title, device manufacturer, drug manufacturer, device trade name, keyword heading word, floating subheading word, candidate term word] 305792

24 limit 23 to (english language and yr="2000") 4825

25 arm.mp. [mp=title, abstract, heading word, drug trade name, original title, device manufacturer, drug manufacturer, device trade name, keyword heading word, floating subheading word, candidate term word] 319379

26 limit 25 to (english language and yr="2000") 4825

27 16 or 18 or 20 or 22 or 24 or 26 11286

28 fracture.mp. [mp=title, abstract, heading word, drug trade name, original title, device manufacturer, drug manufacturer, device trade name, keyword heading word, floating subheading word, candidate term word] 362800

29 limit 26 to (english language and yr="2000") 4454

30 treatment.mp. [mp=title, abstract, heading word, drug trade name, original title, device manufacturer, drug manufacturer, device trade name, keyword heading word, floating subheading word, candidate term word] 7342370

31 limit 30 to (english language and yr="2000") 92159

32 surgical.mp. [mp=title, abstract, heading word, drug trade name, original title, device manufacturer, drug manufacturer, device trade name, keyword heading word, floating subheading word, candidate term word] 1746238

33 limit 32 to (english language and yr="2000") 23028

34 operative.mp. [mp=title, abstract, heading word, drug trade name, original title, device manufacturer, drug manufacturer, device trade name, keyword heading word, floating subheading word, candidate term word] 450690

35 limit 34 to (english language and yr="2000") 4128

36 rehabilitation.mp. [mp=title, abstract, heading word, drug trade name, original title, device manufacturer, drug manufacturer, device trade name, keyword heading word, floating subheading word, candidate term word] 360647

47 limit 36 to (english language and yr="2000") 4773

48 31 or 33 or 35 or 36 108298

49 14 and 27 and 29 and 48: **10 results**

| **1.2 Databases: CINAHL (Plus)**Searched on 20/03/2023 **Limits:** English only, 01-01-2000 to 20-03-2023 | | | | |
| --- | --- | --- | --- | --- |
| **#** | **Query** | **Limiters/Expanders** | **Last Run Via** | **Results** |
| S29 | S8 AND S15 AND S18 AND S23 | Expanders - Apply equivalent subjects Search modes - Boolean/Phrase | Interface - EBSCOhost Research Databases Search Screen - Advanced Search Database - CINAHL Plus | 2,261 |
| S23 | S19 OR S20 OR S21 OR S22 | Expanders - Apply equivalent subjects Search modes - Boolean/Phrase | Interface - EBSCOhost Research Databases Search Screen - Advanced Search Database - CINAHL Plus | 1,494,646 |
| S22 | "rehabilitation" | Limiters - Published Date: 20000101-; Language: English Expanders - Apply equivalent subjects Search modes - Boolean/Phrase | Interface - EBSCOhost Research Databases Search Screen - Advanced Search Database - CINAHL Plus | 170,307 |
| S21 | "operative" | Limiters - Published Date: 20000101-; Language: English Expanders - Apply equivalent subjects Search modes - Boolean/Phrase | Interface - EBSCOhost Research Databases Search Screen - Advanced Search Database - CINAHL Plus | 80,243 |
| S20 | "surgical" | Limiters - Published Date: 20000101-; Language: English Expanders - Apply equivalent subjects Search modes - Boolean/Phrase | Interface - EBSCOhost Research Databases Search Screen - Advanced Search Database - CINAHL Plus | 263,267 |
| S19 | "treatment" | Limiters - Published Date: 20000101-; Language: English Expanders - Apply equivalent subjects Search modes - Boolean/Phrase | Interface - EBSCOhost Research Databases Search Screen - Advanced Search Database - CINAHL Plus | 1,203,144 |
| S18 | S16 OR S17 | Expanders - Apply equivalent subjects Search modes - Boolean/Phrase | Interface - EBSCOhost Research Databases Search Screen - Advanced Search Database - CINAHL Plus | 70,854 |
| S17 | "broken" | Limiters - Published Date: 20000101-; Language: English Expanders - Apply equivalent subjects Search modes - Boolean/Phrase | Interface - EBSCOhost Research Databases Search Screen - Advanced Search Database - CINAHL Plus | 4,370 |
|  |  |  |  |  |
| S16 | "fracture" | Limiters - Published Date: 20000101-; Language: English Expanders - Apply equivalent subjects Search modes - Boolean/Phrase | Interface - EBSCOhost Research Databases Search Screen - Advanced Search Database - CINAHL Plus | 66,836 |
| S15 | S9 OR S10 OR S11 OR S12 OR S13 OR S14 OR S14 | Expanders - Apply equivalent subjects Search modes - Boolean/Phrase | Interface - EBSCOhost Research Databases Search Screen - Advanced Search Database - CINAHL Plus | 121,779 |
| S14 | "arm" | Limiters - Published Date: 20000101-; Language: English Expanders - Apply equivalent subjects Search modes - Boolean/Phrase | Interface - EBSCOhost Research Databases Search Screen - Advanced Search Database - CINAHL Plus | 59130 |
| S13 | "distal" | Limiters - Published Date: 20000101-; Language: English Expanders - Apply equivalent subjects Search modes - Boolean/Phrase | Interface - EBSCOhost Research Databases Search Screen - Advanced Search Database - CINAHL Plus | 36,745 |
| S12 | "midshaft" | Limiters - Published Date: 20000101-; Language: English Expanders - Apply equivalent subjects Search modes - Boolean/Phrase | Interface - EBSCOhost Research Databases Search Screen - Advanced Search Database - CINAHL Plus | 603 |
| S11 | "proximal" | Limiters - Published Date: 20000101-; Language: English Expanders - Apply equivalent subjects Search modes - Boolean/Phrase | Interface - EBSCOhost Research Databases Search Screen - Advanced Search Database - CINAHL Plus | 33,429 |
| S10 | "humeral" | Limiters - Published Date: 20000101-; Language: English Expanders - Apply equivalent subjects Search modes - Boolean/Phrase | Interface - EBSCOhost Research Databases Search Screen - Advanced Search Database - CINAHL Plus | 6,069 |
| S9 | Humerus | Limiters - Published Date: 20000101-; Language: English Expanders - Apply equivalent subjects Search modes - Boolean/Phrase | Interface - EBSCOhost Research Databases Search Screen - Advanced Search Database - CINAHL Plus | 5,514 |
| S8 | S1 OR S2 OR S3 OR S4 OR S5 OR S6 OR S7 | Expanders - Apply equivalent subjects Search modes - Boolean/Phrase | Interface - EBSCOhost Research Databases Search Screen - Advanced Search Database - CINAHL Plus | 646,568 |
| S7 | "immediate" | Limiters - Published Date: 20000101-; Language: English Expanders - Apply equivalent subjects Search modes - Boolean/Phrase | Interface - EBSCOhost Research Databases Search Screen - Advanced Search Database - CINAHL Plus | 43,781 |
| S6 | "patient guided" | Limiters - Published Date: 20000101-; Language: English Expanders - Apply equivalent subjects Search modes - Boolean/Phrase | Interface - EBSCOhost Research Databases Search Screen - Advanced Search Database - CINAHL Plus | 34 |
| S5 | "loading" | Limiters - Published Date: 20000101-; Language: English Expanders - Apply equivalent subjects Search modes - Boolean/Phrase | Interface - EBSCOhost Research Databases Search Screen - Advanced Search Database - CINAHL Plus | 18,472 |
| S4 | "avoid" | Limiters - Published Date: 20000101-; Language: English Expanders - Apply equivalent subjects Search modes - Boolean/Phrase | Interface - EBSCOhost Research Databases Search Screen - Advanced Search Database - CINAHL Plus | 45,287 |
| S3 | "early" | Limiters - Published Date: 20000101-; Language: English Expanders - Apply equivalent subjects Search modes - Boolean/Phrase | Interface - EBSCOhost Research Databases Search Screen - Advanced Search Database - CINAHL Plus | 359,237 |
| S2 | weight bearing | Limiters - Published Date: 20000101-; Language: English Expanders - Apply equivalent subjects Search modes - Boolean/Phrase | Interface - EBSCOhost Research Databases Search Screen - Advanced Search Database - CINAHL Plus | 11,731 |
| S1 | Weight | Limiters - Published Date: 20000101-; Language: English Expanders - Apply equivalent subjects Narrow by Language: - English Search modes - Boolean/Phrase | Interface - EBSCOhost Research Databases Search Screen - Advanced Search Database - CINAHL Plus | 210,113 |

**Results: 2261**

# **2. Website for Grey Literature:**

| **Search location** | **Search (date)** | **Limits** | **Results** |
| --- | --- | --- | --- |
| ClinicalTrials.gov | "weight" OR "weight bearing" OR "immediate" OR "early" OR "avoid" OR "non" OR "loading" OR "patient guided" AND "humerus" OR "humeral" OR "proximal" OR "midshaft" OR "distal" OR "arm" AND "fracture" OR "fractures" OR "fractured" OR "broken" AND "treatment" OR "surgical" OR "non surgical" OR "operative" OR "non operative" OR "rehabilitation" (12/03/23) | Start date: 01/01/2000  Age group: 18-64 and 65+ | 8998 |
| Cochrane Central Register of Controlled Trials (CENTRAL) | (“weight bear*” OR “load*”) AND (“humer*” OR “proximal” OR “midshaft” OR “distal”) AND (“fracture*” OR “broken”) AND (“treatment” OR “surgery” OR “operati*” OR “rehabilitation”) (12/03/23) | Start date: 01/01/2000  English language | 106 |
| NIHR Open Research | ("weight" OR "weight bearing" OR "immediate" OR "early" OR "avoid" OR "non" OR "loading" OR "patient guided") AND ("humerus" OR "humeral" OR "proximal" OR "midshaft" OR "distal" OR "arm") AND ("fracture" OR "fractures" OR "fractured" OR "broken") AND ("treatment" OR "surgical" OR "non surgical" OR "operative" OR "non operative" OR "rehabilitation") (12/03/23) | Not applicable | 237 |
| OpenGrey.EU | (“weight bear*” OR “load*”) AND (“humer*” OR “proximal” OR “midshaft” OR “distal”) AND (“fracture*” OR “broken”) AND (“treatment” OR “surgery” OR “operati*” OR “rehabilitation”) (12/03/23) | Not applicable | 6401 |
