# Supplementary material for: The feasibility, acceptability, safety, and effects of early weight bearing in humeral fractures – a scoping review
Source: Disabil Rehabil. 2024 May 16;47(3):519–30. doi: 10.1080/09638288.2024.2351594 (PMC11789713; doi:10.1080/09638288.2024.2351594)
Supplement: Supplemental Material [file IDRE_A_2351594_SM6464.zip › Records excluded in second full text screening.docx]

**Reason 1:** wrong population (n = 83)

**Reason 2**: wrong intervention (n = 275)

**Reason 3:** reports not retrieved as no full text (n = 1)

**Reason 4:** non-English language (n=1)

**Reason 5:** did not specify weight bearing below 6-weeks (n= 15)

Total = 375 articles

| **Reason 1: wrong population (i.e., involving of biomedical artificial bones, animal, paediatrics below 18-year-old) (n = 83)** |
| --- |
| 1. Adesope et al 2023 2. Atalar et al 2017 3. Bienati et al 2022 4. Bledsoe et al 2021 5. Borbas et al 2021 6. Borbas et al 2022 7. Brais et al 2015 8. Bruder et al 2017 9. Caravaggi et al 2014 10. Catanzarite et al 2009 11. Chen et al 2002 12. Chen et al 2017 13. Choo et al 2005 14. Chudik et al 2003 15. Cruickshank et al 2015 16. Dahan et al 2019 17. Davis et al 2012 18. Dieterich et al 2006 19. Duda et al 2007 20. Esen et al 2009 21. Euler et al 2017 22. Feng et al 2012 23. Fletcher et al 2019 24. Fuchtmeier et al 2007 25. Gomes et al 2021 26. Grunewald et al 2019 27. Gwilym et al 2020 28. Hak et al 2010 29. Hong et al 2018 30. Horn et al 2011 31. Hsiao et al 2017 32. Huang H 2019 33. Ishii et al 2020 34. Jabran et al 2018 35. Kaisidis et al 2018 36. Katthagen et al 2015 37. Katthagen et al 2018 38. Kong et al 2021 39. Konrad et al 2008 40. Kowalska et al 2001 41. Maldonado et al 2003 42. Martinez-Catalan et al 2022 43. Martinez-Catalan et al 2023 44. Mehling et al 2009 45. Patel et al 2011 46. Patel et al 2016 47. Panagiotopoulou et al 2021 48. Pastor et al 2023 49. Penzkofer et al 2010 50. Ponce et al 2013 51. Razaeian et al 2020 52. Reising et al 2014 53. Roderer et al 2007 54. Rose DM et al 2010 55. Rusimov et al 2019 56. Sanders et al 2007 57. Sandmann et al 2020 58. Schmalzl et al 2021 59. Schorler et al 2017 60. Schumer et al 2010 61. Schwartz et al 2006 62. Seppel et al 2017 63. Shah et al 2020 64. Stone A 2023 65. Taylor et al 2016 66. Theopold et al 2018 67. Tilton et al 2020 68. Tingstad et al 2020 69. Varady et al 2017 70. Varga et al 2017 71. Verbruggen et al 2007 72. Vogel et al 2007 73. Voigt et al 2011 74. Wagner et al 2020 75. Walsh et al 2006 76. Wang et al 2013 77. Weeks et al 2013 78. Windolf et al 2010 79. Yamamoto et al 2013 80. Yoon et al 2014 81. Zha et al 2023 82. Ziran et al 2010 83. Zumstein et al 2015 |

| **Reason 2: wrong intervention [did not address the concept of early weight bearing or loading in humeral fractures] (n = 275)** |
| --- |
| 1. Abimanyi-Ochom et al 2015 2. Abzug and Dantuluri 2010 3. Acklin and Sommer 2012 4. Agarwal et al 2006 5. Agorastides et al 2007 6. Aguado et al 2018 7. Aguado et al 2021 8. Ali et al 2005 9. Apivatthakakul et al 2009 10. Aschauer et al 2007 11. Atici et al 2021 12. Atif et al 2019 13. Athwal et al 2009 14. Attala et al 2021 15. Avilucea et al 2020 16. Babhulkar et al 2011 17. Bahman et al 2021 18. Baldairon et al 2023 19. Bandalovic et al 2014 20. Barlow et al 2011 21. Barth et al 2023 22. Bari et al 2004 23. Basa et al 2020 24. Batten et al 2018 25. Ben Fadhel et al 2022 26. Benzinger et al 2019 27. Bercik et al 2012 28. Beredjiklian et al 2002 29. Bhashyam et al 2020 30. Bisaccia et al 2017 31. Blum et al 2009 32. Bones et al 2022 33. Bono et al 2000 34. Brorson et al 2009 35. Bruder et al 2017 36. Burg et al 2011 37. Burton et al 2005 38. Callaghan et al 2017 39. Campochiaro et al 2017 40. Canbora et al 2013 41. Capo et al 2014 42. Carlock et al 2021 43. Celli et al 2008 44. Ceynowa et al 2018 45. Chehab et al 2005 46. Coforio and Maniscalco 2017 47. Cruickshank et al 2015 48. Dahm et al 2022 49. Darabos et al 2012 50. Demir et al 2020 51. Dean et al 2016 52. Dekker et al 2021 53. Denies et al 2010 54. Dimakopoulos et al 2009 55. Durak et al 2022 56. Duquin et al 2014 57. Dwijen et al 2022 58. Eid et al 2011 59. Ekwedigwe et al 2021 60. Elmadag et al 2014 61. Eralp et al 2001 62. Erdogan et al 2014 63. Fakler et al 2008 64. Fu et al 2019 65. Gaheer and Hawkins 2010 66. Gallay et al 2000 67. George et al 2015 68. Giordano et al 2021 69. Green et al 2009 70. Greiner et al 2008 71. Gupta et al 2013 72. Handoll et al 2009 73. Handoll et al 2015a 74. Handoll et al 2015b 75. He et al 2019 76. Hodgson et al 2003 77. Hodgson S 2006 78. James wt al 2014 79. Jain et al 2017 80. Ju et al 2022 81. Kaser et al 2004 82. Keding et al 2019 83. Khattak et al 2019 84. Kilcarsian et al 2011 85. Koljonen 86. Kontakis et a 2009 87. Kose et al 2016 88. Krappinger et al 2011 89. Kruithof et al 2017 90. LaCoste et al 2021 91. Lam et al 2022 92. Launonen et al 2016 93. Launonen et al 2019 94. Lee et al 2013 95. Liaghat et al 2022 96. Mahabier et al 2013 97. Maldobado et al 2003 98. Marchetti et al 2000 99. Maresca et al 2016 100. Marinelli et al 2018 101. Matsunaga et al 2013 102. Matuszewski et al 2015 103. Mavrogenis et al 2011 104. McLaurin TM 2004 105. Mellstrand et al 2018 106. Mocini et al 2020 107. Mohanty et al 2022 108. Muller et al 2005 109. Muzaffar et al 2014 110. Nerz et al 2017 111. Niemeyer et al 2004 112. Nijs et al 2011 113. O’Driscoll et al 2004 114. Orman et al 2020 115. Ozturk et al 2008 116. Panagopoulos et al 2018 117. Pankaj et al 2007 118. Pantalone et al 2017 119. Papakonstantinou et al 2017 120. Pappu et al 2022 121. Park et al 2012 122. Park et al 2014 123. Park et al 2016 124. Parlato et al 2014 125. Parmaksizoglu et al 2010 126. Paryavi et al 2010 127. Pavic et al 2012 128. Pawaskar et al 2012 129. Petsatodes et al 2004 130. Piekarczyk et al 2015 131. Plecko and Kraus 2005 132. Pidhorz et al 2013 133. Piggott et al 2022 134. Plecko et al 2005 135. Polat et al 2009 136. Popovic et al 2012 137. Rabi et al 2015 138. Radoicic et al 2014 139. Razaeian et al 2020 140. Razaeian et al 2023 141. Rehman et al 2000 142. Rebuzzi et al 2010 143. Richetti et al 2010 144. Ring et al 2003 145. Ristic et al 2011 146. Ritter et al 2023 147. Rivero et al 2022 148. Robinson et al 2003a 149. Robinson et al 2003b 150. Robinson et al 2011 151. Roderer et al 2007 152. Roderer et al 2010 153. Rollo et al 2019 154. Rollo et al 2021 155. Rose DM et al 2013 156. Rotman 2020 157. Routman HD 2013 158. Rouleau et al 2009 159. Ruchelsman et al 2008 160. Rudran et al 2022 161. Ruland WO 2000 162. Russo et al 2008 163. Russo et al 2015 164. Sabah et al 2021 165. Sabesan et al 2015 166. Sabharwal et al 2016 167. Sacchetti et al 2020 168. Saragaglia et al 2013 169. Saul et al 2017 170. Salvador et al 2019 171. Samborski et al 2021 172. Samborski et al 2023 173. Sanchez-Sotelo J 2012 174. Savvidou et al 2018 175. Saul et al 2017 176. Schairer et al 2017 177. Scaglione et al 2022 178. Schindelar et al 2019 179. Schliemann et al 2012 180. Schmalz et al 2021 181. Schmidt-Horlohe et al 2013 182. Schoch et al 2017 183. Schumaier et al 2018 184. Schindelar et al 2019 185. Sebatia-Forcada et al 2017 186. Seo et al 2016 187. Seyfettinoglu et al 2018 188. Shannon et al 2018 189. Sharma et al 2011 190. Sharma et al 2020 191. Shen et al 2013 192. Shields et al 2015 193. Shields et al 2016 194. Singh et al 2012 195. Singh AP 2015 196. Singhal et al 2015 197. Siwach et al 2009 198. Slobogean et al 2015 199. Smolle et al 2022 200. Stone et al 2023 201. Sommer et al 2003 202. Song et al 2020 203. Soon et al 2004 204. Sosef et al 2007 205. Souleiman et al 2023 206. Spitzer et al 2009 207. Spross et al 2017 208. Spross et al 2019 209. Spross et al 2021 210. Stamatis and Paxinos 2003 211. Stone et al 2023 212. Strohm et al 2011 213. Suk-Hwan et al 2018 214. Takeuchi et al 2002 215. Tallay et al 2022 216. Tan et al 2012 217. Tan et al 2014 218. Tao et al 2022 219. Tarallo et al 2015 220. Tauber et al 2015 221. Tejwani et al 2008 222. Tennant et al 2002 223. Terragnoli et al 2007 224. Thakur et al 2016 225. Thanasas et al 2009 226. Thelen et al 2022 227. Tomori et al 2022 228. Touloupakis et al 2017 229. Touloupakis et al 2019 230. Trikha et al 2017 231. Tyllianakis et al 2022 232. Uchiyama et al 2022 233. Ueda et al 2014 234. Upadhay and Lil 2017 235. Urda et al 2012 236. Uzer et al 2015 237. Vallier HA 2007 238. Van De Wall et al 2020 239. Van de Water et al 2015 240. Van Middendorp et al 2011 241. Varecka and Myeroff 2017 242. Verbruggen and Stapert 2007 243. Verdano et al 2018 244. Verma et al 2017 245. Vicenti et al 2019 246. Vijayvargiya et al 2016 247. Viswanathan et al 2023 248. Von der Helm et al 2022 249. Von Keudell et al 2016 250. Vora et al 2020 251. Wall et al 2007 252. Walter et al 2023 253. Wang et al 2021 254. Wang et al 2022 255. Wanner et al 2003 256. Walter et al 2023 257. Watson et al 2017 258. Widnall et al 2013 259. Willis and Ahmadi 2019 260. Wolfensperger et al 2017 261. Yadaz er al 2016 262. Yang et al 2003 263. Yian et al 2016 264. Yigit S 2020 265. Yigit et al 2022 266. Yorukoglu et al 2018 267. Young et al 2008 268. Young et al 2010 269. Zachariasen et al2020 270. Zalavras et al 2007 271. Zhang et al 2017 272. Zhang et al 2020 273. Zhe et al 2014 274. Ziegler et al 2019 275. Zingg et al 2002 |

| **Reason 3: not retrieved as no full text (n = 1)** |
| --- |
| 1. Rapala and Obrebski 2003 |

| **Reason 4: non-English (n = 1)** |
| --- |
| 1. Plath JE 2018 |

| **Reason 5: Did not specify weight bearing below 6-weeks [or potential delay weight bearing/loading from 6-weeks onwards, or unspecific timing to commence weight bearing such as waiting for radiographic or clinical healing] (n= 15)** |
| --- |
| 1. Acklin et al 2012 2. Adesope et al 2023 3. Aksoy et al 2010 4. Bienati et al 2022 5. Herteleer et al 2023 6. Hessmann et al 2012 7. Jain et al 2017 8. Mao et al 2022 9. Polykandriotis et al 2021 10. Rouleau et al 2020 11. Shannon et al 2018 12. Singh et al 2014 13. Tao et al 2022 14. Van De Wall et al 2019 15. Ziegler et al 2019 |
